# Bilateral Diaphragmatic Paresis Due to an Iatrogenic Injury of the Phrenic Nerve: A Case Report

**DOI:** 10.7759/cureus.72134

**Published:** 2024-10-22

**Authors:** Teresa Martins, Carla Hovenkamp, Helena Temido, Joana Martins, João Silveira

**Affiliations:** 1 Physical Medicine and Rehabilitation, Unidade Local de Saúde de Coimbra, Coimbra, PRT; 2 Internal Medicine, Unidade Local de Saúde de Coimbra, Coimbra, PRT

**Keywords:** diaphragmatic paresis, dyspnea, phrenic nerve, respiratory insufficiency, respiratory paralysis

## Abstract

Bilateral diaphragmatic paresis is a rare entity with unknown incidence and is associated with severe respiratory dysfunction. In this case report we present a 69-year-old patient who sought emergency services due to dyspnea, orthopnea, and paroxysmal nocturnal dyspnea. The patient denied other complaints and mentioned that these symptoms began after being discharged from a hospitalisation for emphysematous acute cholecystitis, where he underwent laparoscopic cholecystectomy, which was complicated by extensive hemorrhage of the abdominal wall. On the physical examination, paradoxical breathing and peripheral oxygen saturation between 80-93% in supine and standing positions, respectively, were notable. The patient was admitted for further investigation, during which thoracic radiographs in inspiration and expiration showed no positional variation of the diaphragm, respiratory function tests revealed a restrictive pattern and electromyography demonstrated acute bilateral diaphragmatic neuropathic injury compatible with phrenic nerve injury. Therefore, by temporal correlation and exclusion of other apparent causes, an etiology in the context of the cholecystectomy was inferred. The patient was evaluated in a multidisciplinary manner and is currently undergoing a cardiopulmonary rehabilitation program in the Physical Medicine and Rehabilitation service, although with limited progress and still requiring non-invasive ventilation.

## Introduction

The diaphragm is the primary respiratory muscle. It is composed of two halves. It is innervated by the phrenic nerve, which originates from the spinal roots C3-C5. Thus, bilateral diaphragmatic paresis is a rare entity with unknown incidence and associated with severe respiratory dysfunction since, in diaphragmatic injuries, inspiration is compromised due to the absence or impairment of the caudal movement of the diaphragm. This causes a limitation in thoracic expansion, resulting in hypoventilation. This entity has various etiologies, with phrenic nerve injury associated with thoracic surgeries being the most common. Therefore, diaphragmatic paresis can be associated with trauma, surgical complications, infectious pathology, or neurological changes [1,2]. It usually presents suddenly, with orthopnea, exercise intolerance, and paradoxical breathing [1,3]. Its diagnosis may include several complementary methods, including thoracic radiographs in inspiration and expiration, arterial blood gas analysis, respiratory function tests, electromyography, and possibly fluoroscopy and direct measurement of transdiaphragmatic pressure [1]. The treatment of diaphragmatic paresis depends on its cause. Options may include diaphragmatic repair surgery or a non-invasive approach [2,4-5]. Being a rare entity and mainly associated with thoracic surgeries, its diagnosis in other contexts is demanding and its management is complex.

## Case presentation

Patient information

A 69-year-old caucasian male patient went to the emergency service due to dyspnea, orthopnea, and paroxysmal nocturnal dyspnea that had been progressing for two weeks. He denied a history of cough, fever, chest pain, lower limb edema, and any history of decreased urine output. The symptoms began two days after being discharged from a General Surgery ward, where he had been admitted for acute emphysematous cholecystitis and had undergone laparoscopic cholecystectomy, complicated by extensive hemorrhage of the abdominal wall. His medical history included well-controlled diabetes mellitus, essential hypertension, and dyslipidemia. The patient was chronically medicated with dapagliflozin 10 mg daily, gliclazide 60 mg daily, metformin + vildagliptin 1000 mg + 50 mg twice daily, indapamide + amlodipine 1.5 mg + 10 mg daily and simvastatin + ezetimibe 10 mg + 10 mg daily.

Clinical findings

**Figure 1:**
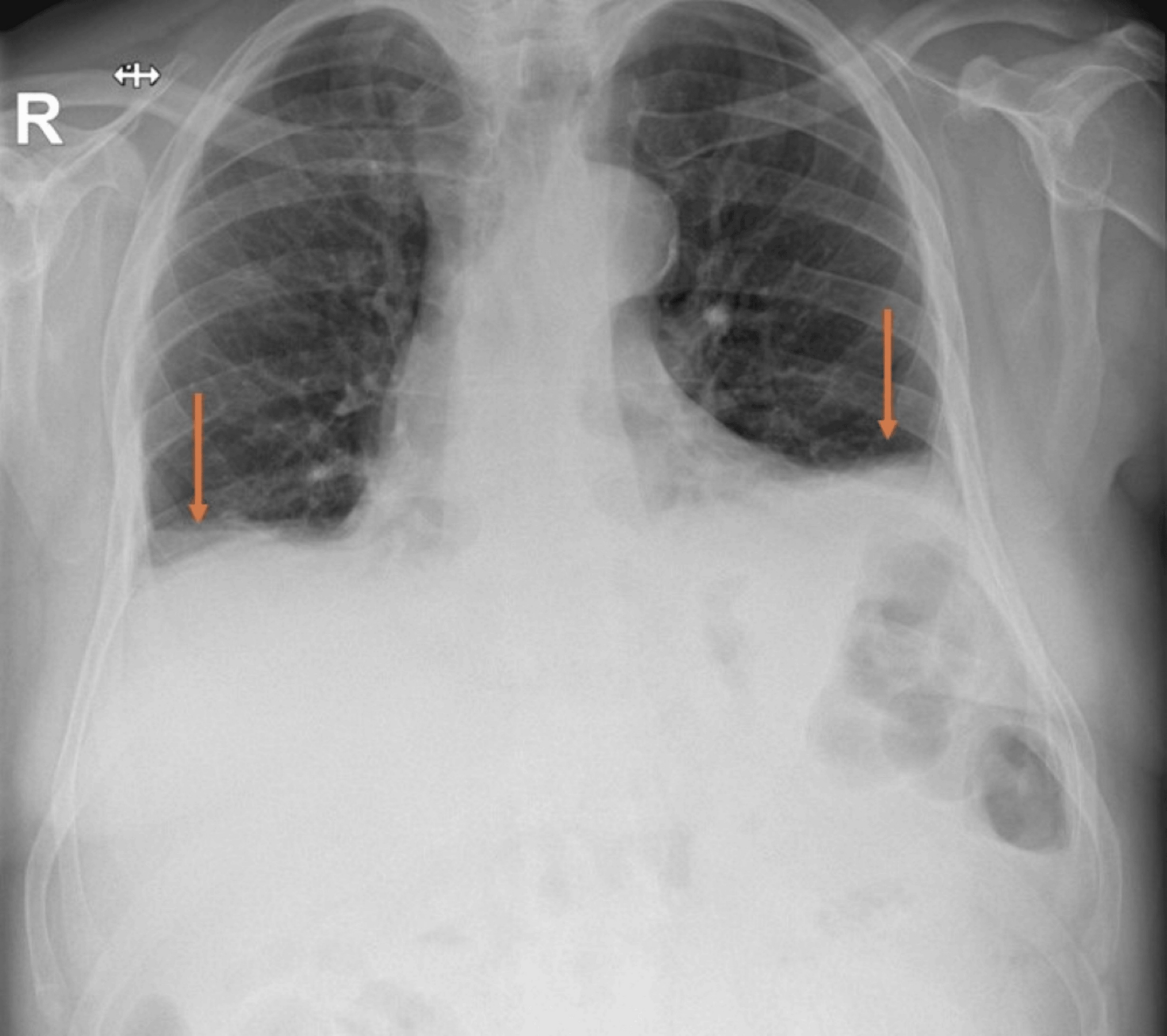
Blunting of both costophrenic angles Arrows indicate bilateral opacification of costophrenic angles that was initially interpreted as pleural effusion in the setting of cardiac failure.

He was hemodynamically stable but exhibited tachypnea, paradoxical breathing, and globally reduced breath sounds. There was no evidence of elevated jugular venous pressure. His peripheral oxygen saturation fluctuated between 80-93% in supine and standing positions. From the examination conducted in the emergency service, he showed global respiratory failure (type II) on arterial blood gas analysis (pH 7.43, pCO_2_ 49 mmHg, pO_2_ 54 mmHg, HCO_3_- 32.5 mEq/L, SatO_2_ 89%, Lactate 0.91 mmol/L), but did not present further significant laboratory changes. He underwent a chest radiography, which showed an apparent bilateral pleural effusion, and was admitted with a diagnosis of congestive heart failure (Figure 1). Therefore, the patient was admitted to the Internal Medicine service.

Investigation

**Figure 2:**
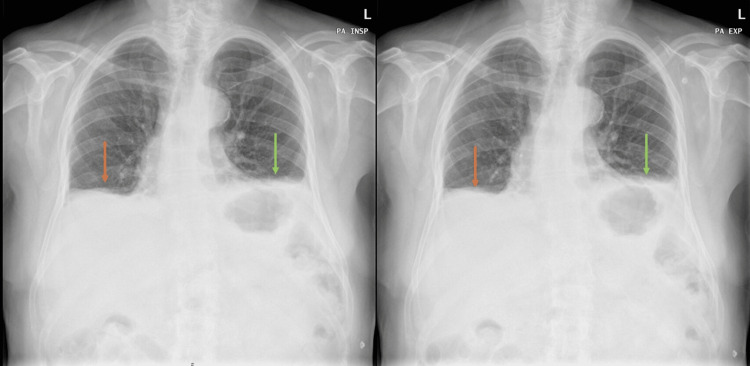
Chest radiographs during inspiration and expiration Absence of positional variation of the diaphragm on chest radiographs during inspiration (left) and expiration (right). The colored arrows demonstrate the absence of positional variation of the diaphragm.

During hospitalization, he remained without significant laboratory changes but continued to experience sudden orthopnea associated with significant desaturation (80%) in the supine position, which was reversed upon standing. Chest radiographs taken during inspiration and expiration showed no positional variation of the diaphragm (Figure 2).

**Figure 3:**
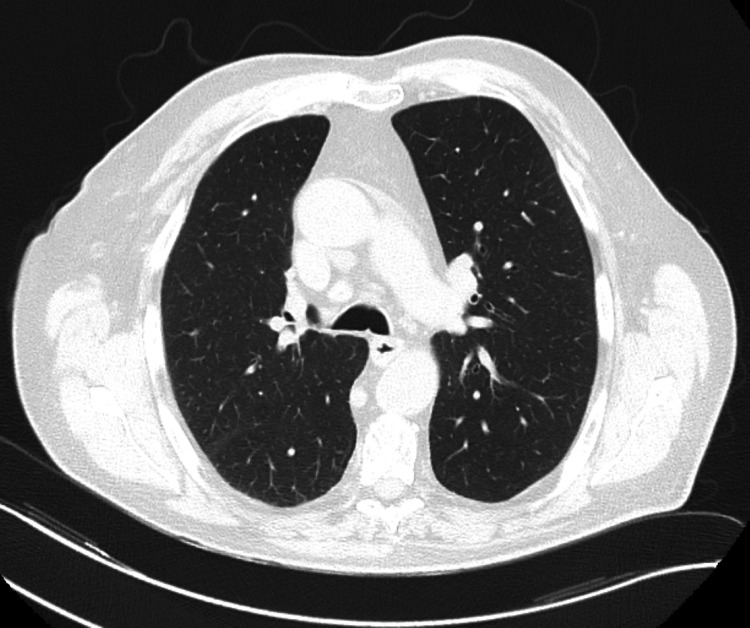
Thoracic computer tomography Absence of significant parenchymal findings that could justify the clinical setting.

**Figure 4:**
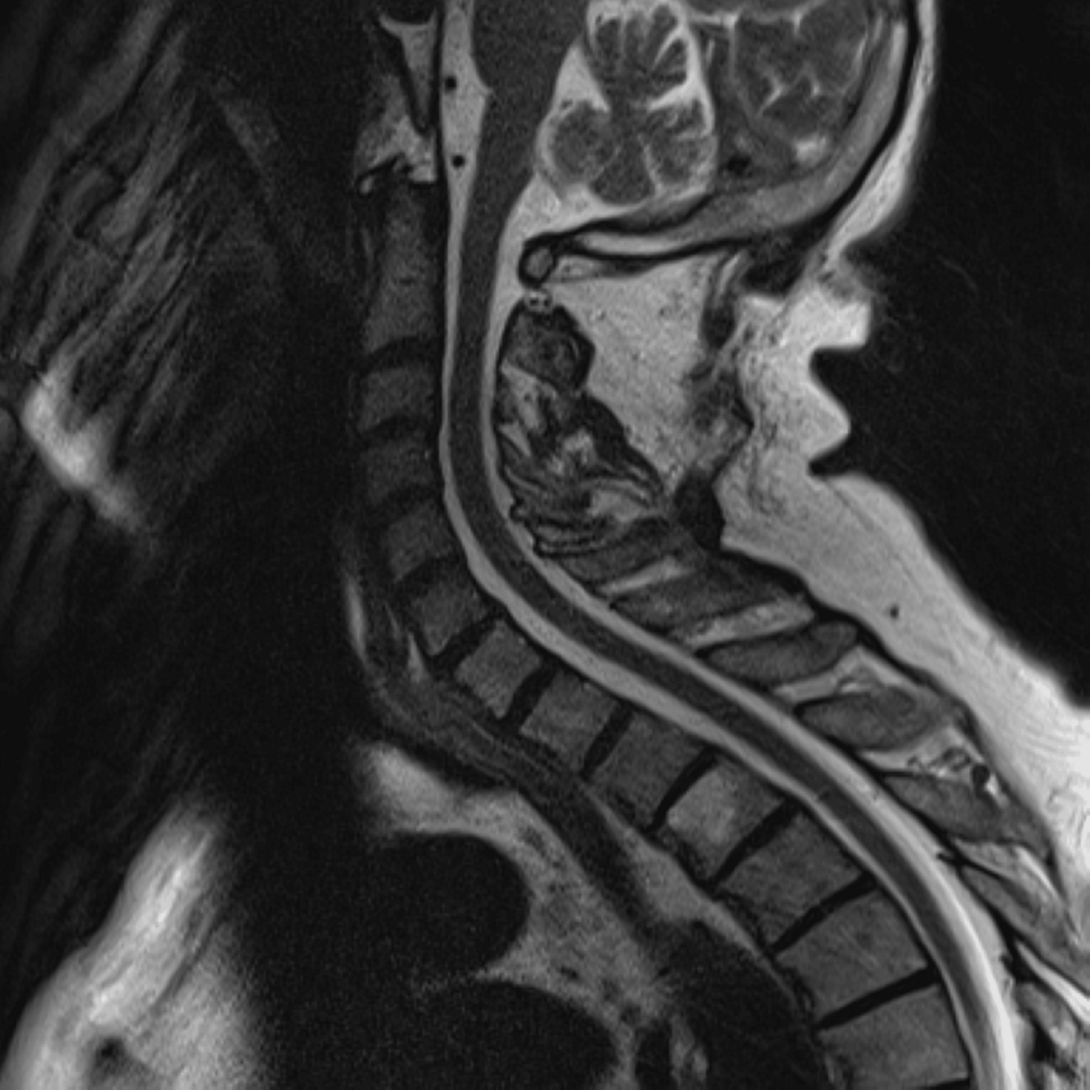
Cervical magnetic resonance imaging Absence of compressive discopathy, focal lesions or reduction in the vertebral canal that would justify the clinical setting.

The respiratory function tests, which were requested in sitting and supine positions (with the patient only tolerating their performance in the sitting position), showed a restrictive pattern, and electromyography revealed acute bilateral neuropathic injury in the diaphragm compatible with phrenic nerve damage. The thoracic computed tomography scan did not show additional changes, and the magnetic resonance imaging of the cervical spine did not reveal any lesions (Figures 3, 4). Therefore, in relation to the timeline, the injury was inferred to be iatrogenic in the context of the cholecystectomy.

Management

The patient was evaluated by thoracic surgery experts for potential surgical correction and was referred for consultations in neurology, pulmonology, and physical medicine and rehabilitation. He underwent a cardiopulmonary rehabilitation program with three weekly sessions, which included training of the respiratory muscles, optimization of ventilatory dynamics in different positions, and low-intensity aerobic training directed at improving symptoms and exercise tolerance. He required Bi-level Positive Airway Pressure (BiPAP) in the supine position since hospital discharge.

**Figure 5:**
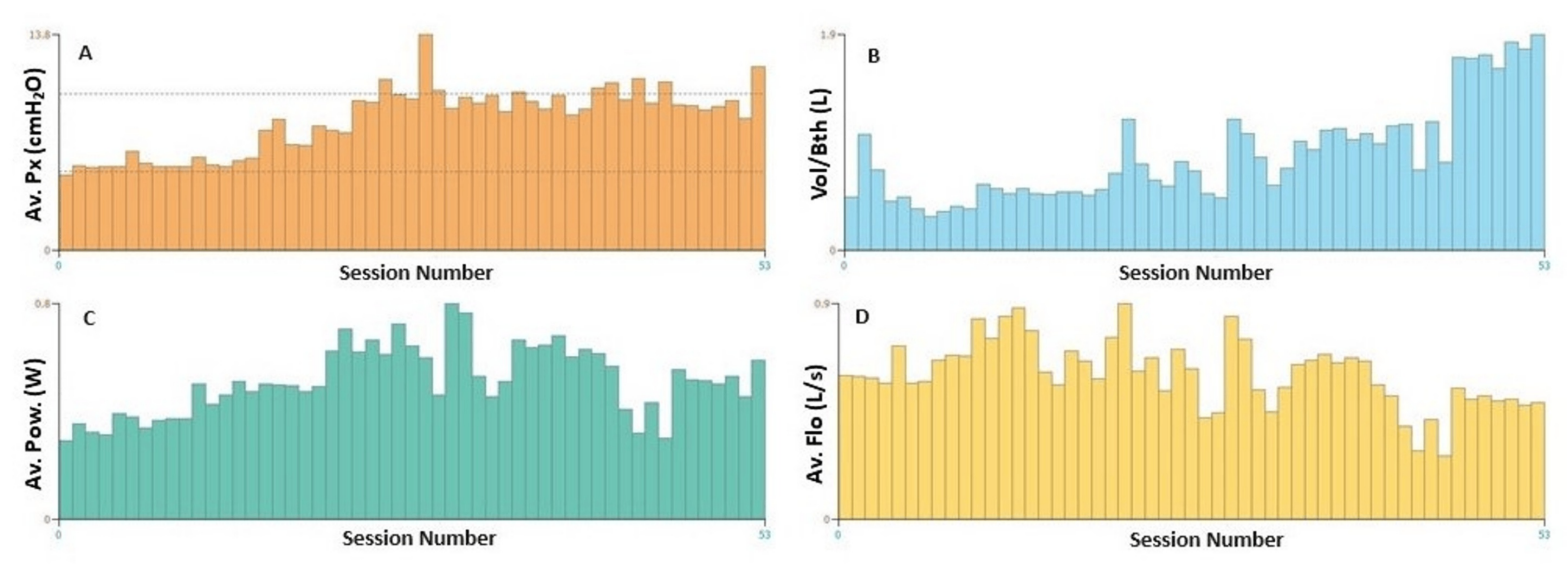
Patient evolution with inspiratory muscle training with POWERbreathe® A: Average inspiratory pressure throughout the sessions; B: Volume mobilized per respiratory cycle throughout the sessions; C: Capacity/Power for inspiratory work throughout the sessions; D: Average flow per respiratory cycle throughout the sessions. POWERbreathe® (POWERbreathe International Ltd., Warwickshire, UK).

At six months of follow-up, he showed significant improvement in exercise tolerance and intensity of dyspnea during exertion in the standing position. However, there was little progress in the respiratory function tests, and the patient still showed prominent symptoms in the supine position, with decreased peripheral oxygen saturation and immediate dyspnea. There was also insignificant progress in the training of the inspiratory muscles with POWERbreathe® (POWERbreathe International Ltd., Warwickshire, UK), evaluated by the parameters: average inspiratory pressure over the sessions (A); volume mobilized per respiratory cycle over the sessions (B); inspiratory work capacity over the sessions (C); average flow per respiratory cycle over the sessions (D) (Figure 5).

## Discussion

Bilateral diaphragmatic paresis is a rare entity with an unknown incidence due to its various possible etiologies, making its diagnosis complex [1]. Several diagnostic tools contribute to the diagnosis of this pathology, including imaging tests such as chest radiography, fluoroscopy, and ultrasound. Respiratory function tests are also performed, which in the case of bilateral diaphragmatic paresis, reveal the presence of a restrictive pattern, with decreased total lung capacity and residual volume, since the diaphragm is especially responsible for inspiration [1]. Fluoroscopy was the gold-standard complementary method for diagnosing this pathology in the past. However, in cases of bilateral diaphragmatic paresis, its interpretation can raise doubts, as these patients may develop altered respiratory patterns to compensate for their difficulties. Currently, the gold-standard method is the direct measurement of transdiaphragmatic pressure, but, being an invasive technique, its use is infrequent [1].

In the clinical case described, the diagnosis was especially complex because, although bilateral diaphragmatic paresis is described as a complication of thoracic surgeries, its association with abdominal surgeries, especially in cases of laparoscopy, is rarer. Thus, it was inferred that the injury was in the context of the cholecystectomy due to its temporal correlation and the exclusion of other possible causes.

For its treatment, both surgical and non-surgical options are available. The choice of therapeutic option depends on the cause of the paresis, the symptoms and the response to previous treatments, as sometimes there is spontaneous resolution or significant improvement of the complaints [1,2]. Among the surgical options, diaphragmatic plication stands out. It involves repositioning the diaphragm in a position of maximum inspiration to allow lung expansion. This treatment is indicated in patients with symptomatic bilateral diaphragmatic paresis who do not experience spontaneous resolution of complaints after a period of six to 12 months [1,6]. Diaphragmatic pacing is a more recent therapeutic option reserved for symptomatic patients dependent on ventilation, aiming to provoke diaphragmatic contraction. It has proven to be very effective, especially when used in combination with diaphragmatic plication. However, for the technique's efficacy, the integrity of the phrenic nerve is required, so it is not applicable in situations where there is damage to the nerve [7,8].

Currently, non-invasive ventilation, especially in BiPAP mode, represents the most important non-invasive therapeutic method in managing symptomatic patients with bilateral diaphragmatic paresis and has shown long-term symptomatic benefit [2,9]. Training of the inspiratory muscles and cardiorespiratory training have also proven essential in increasing muscle strength and symptomatic and functional improvement. However, there is still insufficient data to conclude that this allows patients to wean off ventilator dependence earlier [10].

Despite showing significant self-reported improvement in exercise tolerance, the patient described maintains a slow evolution of respiratory function tests and symptoms in the supine position. Training the respiratory muscles has also shown little significant progress, so other therapeutic approaches may be necessary.

## Conclusions

Bilateral diaphragmatic paresis is a rare entity, and its diagnosis and management are potentially complex. It can evolve with respiratory failure and is potentially life-threatening. This case report demonstrates the association between upper abdominal surgery and bilateral diaphragmatic paresis, the gasometric, respiratory function tests, and imagiologic findings commonly associated with this entity, and the relevance of a multidisciplinary approach that includes a cardiopulmonary rehabilitation program. Furthermore, it highlights the imperative for continuous investigation of treatment and management options since many patients require chronic non-invasive ventilation.
